# Long-Term Complications of Proctectomy for Refractory Perianal Crohn’s Disease: A Narrative Review

**DOI:** 10.3390/jcm14082802

**Published:** 2025-04-18

**Authors:** Bruno Augusto Alves Martins, Mariana Trotta Villar, Luna Vitória Gondim Ferreira, Beatriz da Costa Rossi Ramos de Carvalho, Nicolas Avellaneda, João Batista de Sousa

**Affiliations:** 1 Department of Colorectal Surgery, Hospital Universitário de Brasília, Federal District, Brasilia 70330-750, Brazil; 2 Medical Sciences Postgraduate Program, School of Medicine, University of Brasilia, Federal District, Brasilia 70910-900, Brazil; 3 Department of General Surgery and Academic Investigations Unit, CEMIC University Hospital, Buenos Aires C1430EFA, Argentina

**Keywords:** Crohn’s disease, inflammatory bowel disease, proctectomy, anal fistula, postoperative complications, surgical stomas

## Abstract

Despite a combination of medical and surgical treatments, many patients with perianal Crohn’s disease (CD) continue to experience refractory disease, requiring proctectomy or proctocolectomy, with the creation of a permanent stoma. Although proctectomy is seen as an ultimate treatment aimed at effectively relieving debilitating symptoms and enhancing quality of life, many patients may still face long-term and chronic complications. This narrative review aims to provide an overview of the main complications that patients undergoing proctectomy for CD may experience throughout their lives. Relevant publications addressing complications of proctectomy for refractory perianal CD were searched in the Medline/PubMed, Embase, Cochrane, and LILACS databases. The main long-term complications that patients encounter are related to impaired perineal wound healing, stoma-related issues, sexual and urinary dysfunction, small bowel obstructions, and CD recurrence. These complications negatively affect the quality of life and frequently necessitate further treatment. Patients should receive preoperative counselling regarding the implications of these particular issues, and regular follow-up must be guaranteed to identify any problems early, allowing for prompt treatment.

## 1. Introduction

Crohn’s disease (CD) is an immune-mediated, chronic condition that involves recurring periods of inflammation in the gastrointestinal tract, leading to bowel damage and disability. The entire gastrointestinal tract can be impacted, with the terminal ileum and colon being the most frequently involved. Inflammation tends to be segmental, asymmetrical, and transmural. While most patients are diagnosed with an inflammatory phenotype, complications such as strictures, fistulas, or abscesses arise over time in about fifty percent of patients, often necessitating surgical intervention [1].

About 40% of individuals with CD present symptomatic perianal disease [2]. Perianal fistulas are the main type of anal involvement, with incidence rates of approximately 30%, followed by abscesses, fissures/ulcers, skin tags, and strictures [2]. Perianal CD is predictive of severe disease course [3]. The chronic and disabling symptoms such as pain, discomfort, discharge, and fecal incontinence result in a significant impact on quality of life [4]. Patients end up experiencing frequent hospitalisations and psychological, sexual, social, and work-related problems [4].

Treatment often requires multimodal therapy, with a collaborative effort between gastroenterologists and colorectal surgeons. Nearly 90% of patients with perianal fistulizing CD need surgical treatment, often requiring multiple procedures [5]. The specific type of surgery performed depends on factors such as the presence of proctitis, anal canal ulcers or stenosis, scarring from previous surgeries, and the number and location of fistula tracts [5]. Despite a combination of medical and surgical treatments, many patients continue to experience refractory disease, with around 20% requiring proctectomy or proctocolectomy, with the creation of a permanent stoma [6]. It is not surprising that the optimal treatment strategy for perianal CD and the individual factors that determine this are regarded as one of the top ten research questions in the treatment of inflammatory bowel disease (IBD) [7].

Although proctectomy is seen as an ultimate treatment aimed at effectively relieving debilitating symptoms and enhancing quality of life, many patients may still face long-term and chronic complications. Recently, a new classification of perianal fistulising CD has determined that Class 4 includes patients who experience persistent perineal symptoms following proctectomy [4]. However, long-term complications following proctectomy are not limited to perianal symptoms and may encompass a broad range of manifestations. This narrative review aims to provide an overview of the main complications that patients undergoing proctectomy for CD may experience throughout their lives.

## 2. Materials and Methods

We searched for relevant publications addressing long-term complications of proctectomy for refractory perianal CD using the Medline/PubMed, Embase, Cochrane, and LILACS databases up to 1 April 2025. The following Medical Subject Heading [MeSH] terms alone or matched with the Boolean operators ‘AND’ or ‘OR’ were used: ‘Crohn’s Disease’, ‘perianal disease’, ‘fistulising disease’, ‘proctectomy’, and ‘complications’.

Study types included randomized clinical trials, prospective and retrospective observational studies, case series, case reports, systematic or narrative reviews, and guidelines of scientific societies. The exclusion criteria were the following: (1) video vignettes, (2) animal studies, and (3) non-available full-text articles. No specific language restrictions were applied. The reference lists from the selected studies were reviewed to identify any additional relevant studies. No ethical approval was obtained for this review since the included data were retained from published reports.

Overall, 1024 articles were identified with initial screening. A total of 220 duplicate articles were removed. The titles and abstracts of articles were collected using the search strategy and were reviewed by the authors to identify studies that could potentially align with the study’s objectives. Articles that did not meet the specified search criteria were excluded. The full texts of 87 potentially eligible studies were then obtained and assessed for relevance. Finally, 71 articles were included in this review.

We divided the narrative report into the following topics: perineal wound complications, stoma-related complications, sexual and urinary dysfunction, small bowel obstructions, and CD recurrence.

## 3. Perineal Wound Complications

Impaired postoperative perineal wound healing poses a particular challenge for the medical team and patients undergoing proctectomy. This troublesome complication can lead to chronic discharge of purulent material, pain, and occasional bleeding, adversely impacting patients’ quality of life and often requiring repeated surgical procedures. While this issue can arise in patients with rectal cancer or familial adenomatous polyposis, it is more commonly observed in individuals with inflammatory bowel diseases (IBDs), particularly CD [8].

**Figure 1:**
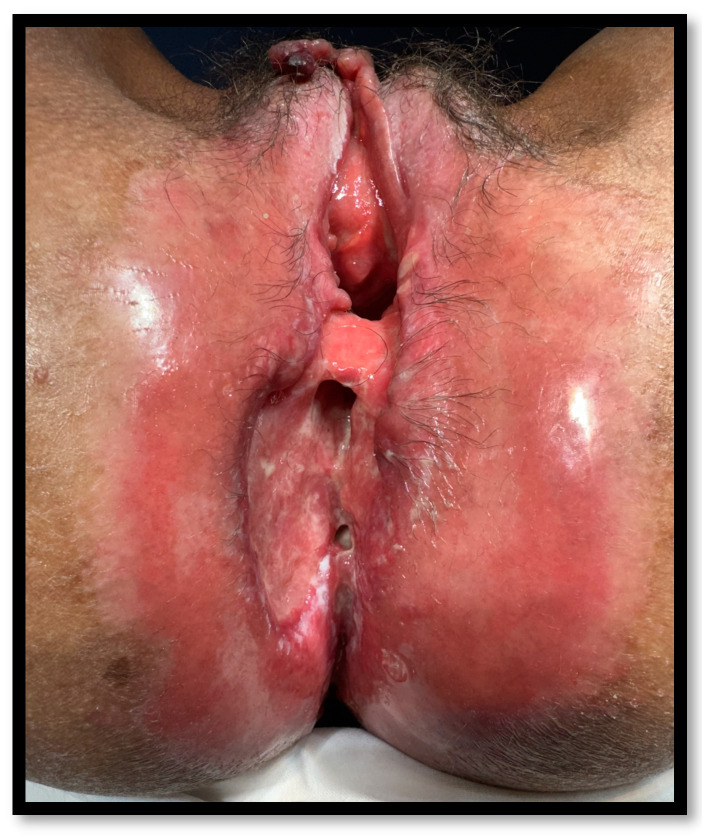
Chronic perineal sinus following proctocolectomy with end ileostomy. The image was taken during the clinical practice of the first author, Mr. Alves Martins, and an informed consent statement for the publication of the photograph was obtained from the patient.

The manifestations of impaired healing can range from superficial wound dehiscence to deep abscesses and long presacral tracts [9,10] (see Figure 1). A chronic perineal sinus (CPS) is a perineal wound that persists for more than 6 months following proctectomy [11]. The reported incidence of CPS in patients undergoing proctectomy for IBD can be as high as 70% [8]. Typically, those sinuses present at the perineal examination as a long fibrous tract covered by infected granulation tissue and an external opening [11].

The exact mechanisms that lead to impaired wound healing in these patients are still not fully understood. Pelvic or perineal sepsis, male gender, younger age, fecal contamination, and rectal involvement have been identified as factors associated with poor healing [9,12,13,14]. A study at the Cleveland Clinic Florida involving data from 136 patients with CD who underwent proctectomy showed a perineal non-healing rate of 21.3% [9]. The only factor linked to delayed healing and non-healing was preoperative sepsis [9]. Grant and collaborators (2020) reviewed data from 103 patients with perianal CD who underwent proctectomy, and almost 40% had a failure of wound healing at 12 months post proctectomy. Male gender was the only variable associated with poor wound healing [13]. Yamamoto and collaborators (1999) analyzed data from 145 patients with CD who underwent proctocolectomy. Multivariate analysis identified younger age, rectal involvement, and fecal contamination at the time of surgery as independent risk factors for perineal sinus [14].

These findings highlight the necessity of a personalized approach that combines the management of preoperative complications with technical considerations to enhance wound healing. Clinical optimization is key to improving outcomes, and preoperative measures such as control of chronic conditions, correction of anemia, nutritional interventions, smoking cessation, steroid tapering, and control of local sepsis must be addressed [10,11]. Control of perineal sepsis before resection with drainage of abscesses and placement of setons is mandatory, and fecal diversion may be considered to reduce perineal inflammation and contamination [10].

Surgeons must be aware of the technical aspects of proctectomy in the context of IBD, as it differs from the conventional abdominoperineal resection for cancer. This is particularly important when addressing extrasphincteric versus intersphincteric dissection. Yamamoto and collaborators (1999) demonstrated that extrasphincteric excision is associated with a greater risk of CPS [14]. Careful hemostasis, debridement, and curettage of chronic inflammatory tissue are also essential to decrease the risk of infection [10]. Regarding mesorectal excision versus close rectal dissection, de Groof and collaborators (2018) have associated mesorectum preservation with higher rates of postoperative perineal complications [15]. Additionally, filling the empty pelvis with omental flaps may be beneficial, using fluorescence angiography with indocyanine green to assess omental perfusion [11,16].

When it comes to treating CPS, the options range from curettage and debridement of necrotic tissue to more complex procedures such as reconstructive surgeries [8,10,11]. The rectus abdominis myocutaneous flap, gracilis muscle transposition, Karydakis flap, cleft closure, and omentoplasty have been described as possible options for treating CPS, presenting an adequate safety profile and reasonable healing rates [16,17,18,19,20,21,22,23]. Adjuvant therapies, including hyperbaric oxygen therapy and negative pressure wound treatment, may play a role in the postoperative closure of perineal wounds [24,25,26,27]. Chan and collaborators (2013) reported their experience using preoperative hyperbaric oxygen therapy in conjunction with rectus abdominis myocutaneous flap repair in four patients with CPS. This approach resulted in complete perineal healing for all patients [24].

Aside from CPS, it is important to note primary CD manifestations that can lead to perineal problems. Perianal CD encompasses non-fistulizing lesions, such as cavitating ulcers, with an incidence of 5–10% [28]. Those ulcerations generally present a prominent margin and an inflammatory aspect. Pain is often severe and unremitting. They may be non-commissural, multiple and extend below the anal margin, through the perineum. Although medical treatment is the primary approach for perianal CD ulcerations, proctectomy may be necessary if medical treatment fails. However, ulcerations can persist or recur even following proctectomy, generating considerable impairment to quality of life [29,30].

Cutaneous Crohn’s disease (CCD) is another condition that can lead to perineal problems following proctectomy [31,32,33]. It is a rare CD extraintestinal manifestation characterized by granulomatous lesions that are non-contiguous with the gastrointestinal tract. CCD lesions can present as plaques, nodules, fistulas, and ulcers, with a particular preference for skin folds. The primary emphasis in diagnosis is the histopathological examination of skin biopsies [34]. Histological features are similar to those encountered in the gastrointestinal tract, with the hallmark finding being inflammatory infiltration characterized by sterile, non-caseating sarcoid-like granulomas, accompanied by an abundance of multinucleated giant cells and plasma cells in the dermis [35]. CCD management is challenging and requires a multidisciplinary approach, encompassing dermatology, gastroenterology, and colorectal surgery. Treatment options include systemic and topical steroids, immunomodulators, and biologics [35]. Surgical debridement and hyperbaric oxygen therapy may be applied in some cases; however, evidence on the issue is scarce [26,35,36].

The malignancy of chronic perineal wounds is a rare yet serious complication. The incidence of malignancy in CPS remains unknown, but in patients with fistulizing perianal CD, the incidence of perianal fistula-related cancer is about 0.8% [37]. The process of carcinogenesis in a non-healing wound can be linked to constant inflammation and trauma, which lead to sustained epithelial regeneration, tissue hypovascularity, and chronic infection. Immunosuppression is also a risk factor for malignant transformation, with persistent symptoms such as pain and changes in wound appearance serving as indicators for careful monitoring and regular biopsies [38,39,40].

## 4. Stoma-Related Complications

An ileostomy or colostomy can significantly enhance a patient’s quality of life. Although it may be straightforward to construct in some patients, if performed outside of ideal conditions and without appropriate planning and consideration of technical and anatomical aspects, it can lead to a range of life-altering complications, affecting both physical and mental well-being [41]. Sadly, stoma creation is linked to considerable morbidity, with high rates of complications occurring both early and later on [42]. Stoma-related complications occur at rates between 20% and 70%. Obesity, emergency surgery, and lack of preoperative marking are associated with increased risk of complications [42]. Early complications encompass ischemia or necrosis, retraction, mucocutaneous separation, and parastomal abscesses. Late complications consist of parastomal hernias, prolapses, and retraction [42].

Parastomal hernias are the most common complications associated with ostomies, occurring in nearly 60% of cases and frequently necessitating surgery [41]. Factors that increase the likelihood of parastomal hernias include obesity, weakness in the abdominal wall, collagen disorders, steroid use, postoperative abdominal sepsis, a large fascial opening (over 3 cm), and any conditions that raise intra-abdominal pressure [43]. While urgent repairs are required for serious issues like incarceration or strangulation, elective repair is recommended for patients experiencing symptoms like intermittent obstruction, incarceration, pain, or trouble keeping the appliance secure on the stoma, even with the use of conservative methods such as ostomy belts, filler rings, and adhesives [41].

Stomal prolapse is full-thickness protrusion of bowel through a stoma. While this complication can arise with any type of ostomy, it is more frequently observed with colostomies than with ileostomies, particularly in loop colostomies of the transverse colon, where it occurs in up to 42% of cases [41,43]. Some risk factors commonly associated with prolapse development include advanced age, obesity, abdominal wall fragility, a large fascial opening, bowel obstruction at the time of stoma creation, redundancy of proximate bowel segments, and conditions that increase intra-abdominal pressure such as chronic cough, ascites, or constipation [41,43]. Elective surgical treatment is primarily indicated for symptomatic patients experiencing pain, skin irritation, or difficulty maintaining an appliance. Emergency surgical indications include incarceration, obstruction, or strangulation of the prolapsed segment [41,42].

Stomal retraction occurs in up to 30% of the cases and is often associated with additional complications such as leakage, peristomal skin irritation, mucocutaneous separations, and peristomal abscess [42,43]. This arises from excessive tension on the stoma, usually secondary to inadequate mobilisation of the intestine. If left untreated, it can lead to stenosis, making ostomy care more difficult [41,43]. Retracted stomas can be primarily managed using convex appliance systems that compress the peristomal skin, thereby enhancing the interface between the appliance and the skin. Additional ostomy accessories, such as belts and binders, may also be utilized. However, if leakage and hygiene problems continue despite these interventions or if stenosis is present, surgical revision should be considered [43].

In addition to the traditional long-term complications associated with stomas, such as parastomal hernia, prolapse, and retraction, specific issues can arise for patients with CD. Those encompass recurrent CD, pyoderma gangrenosum, peristomal fistulae, ulcerations, strictures, granulomas, and peristomal abscesses [41,44].

For those undergoing proctectomy, one particularly concerning complication is the recurrence of CD around the stoma site. Peristomal recurrence can result in skin irritation, dermatitis, nonhealing ulcers, increased stoma output, fistulas, abscesses, stomal retraction, and strictures. Often, these patients need surgical intervention to revise the stoma, with or without additional bowel resections [41,45].

When patients with medically refractory anorectal Crohn’s disease require definitive surgery that results in a permanent stoma, there is some disagreement on whether to perform an isolated proctectomy with an end colostomy or a proctocolectomy with an end ileostomy. This debate stems from the unclear likelihood of disease recurrence in the remaining colon [46]. The reasons for opting to preserve part of the colon and to choose a colostomy over an ileostomy include significantly lower stoma output, which helps avoid dehydration, a lower risk of issues related to peristomal skin, and the assumption of better quality of life [47]. On the other hand, historically, end colostomy has been associated with a higher rate of disease recurrence [48]. De Buck van Overstraeten and colleagues, retrospectively, analyzed ten consecutive patients with refractory distal and perianal CD who underwent intersphincteric proctectomy with end-colostomy. Recurrence in the proximal colon was noted in nine patients, and completion colectomy was necessary in 50% of the patients [47]. Conversely, Lightner and colleagues analyzed data from 63 patients who underwent proctectomy with end colostomy for distal CD, reporting that approximately 29% of the patients experienced endoscopic colonic recurrence and nearly 5% required a completion total abdominal colectomy [46].

Parastomal pyoderma gangrenosum is another complication that may arise in CD patients following proctectomy or proctocolectomy [49]. This condition is marked by a painful superficial ulcer with a clearly defined red border, typically indicating persistent inflammation [41]. Angriman and collaborators (2022) reported follow-up data from a cohort of 54 consecutive patients who underwent surgery for CD involving the creation of any type of stoma, and the incidence rate of pyoderma gangrenosum was approximately 5.5% [44]. Iesalnieks (2022) [49] analyzed outcomes from 99 patients with CD undergoing intestinal resection that included the formation of an ostomy. Ten percent developed peristomal pyoderma gangrenosum, all in the presence of an ileostomy. Non-stricturing and non-penetrating disease, along with preoperative intake of biologics, were identified as risk factors for developing peristomal pyoderma gangrenosum during the postoperative period [49]. In addition to wound care and managing persistent underlying inflammation, the first-line treatment options that have the most robust evidence include systemic corticosteroids, cyclosporine, and tumor necrosis factor-alpha inhibitors [41,50,51].

Surgeons must have a comprehensive understanding of stoma complications and their management. A meticulous approach to creating a stoma, particularly in the context of permanent stomas, can significantly reduce the associated morbidity of this procedure. 

## 5. Small Bowel Obstructions

Colorectal surgery has the highest rate of readmissions related to adhesions among all abdominal surgeries [52]. These procedures carry a 30% risk of adhesion-related complications within four years [52]. Approximately one-third of individuals who develop adhesional obstruction do so within one year of surgery, and up to 18% of patients with adhesional bowel obstruction require reoperation with adhesiolysis [53]. Consequently, this issue significantly impacts the quality of life for patients undergoing colorectal surgery.

**Figure 2:**
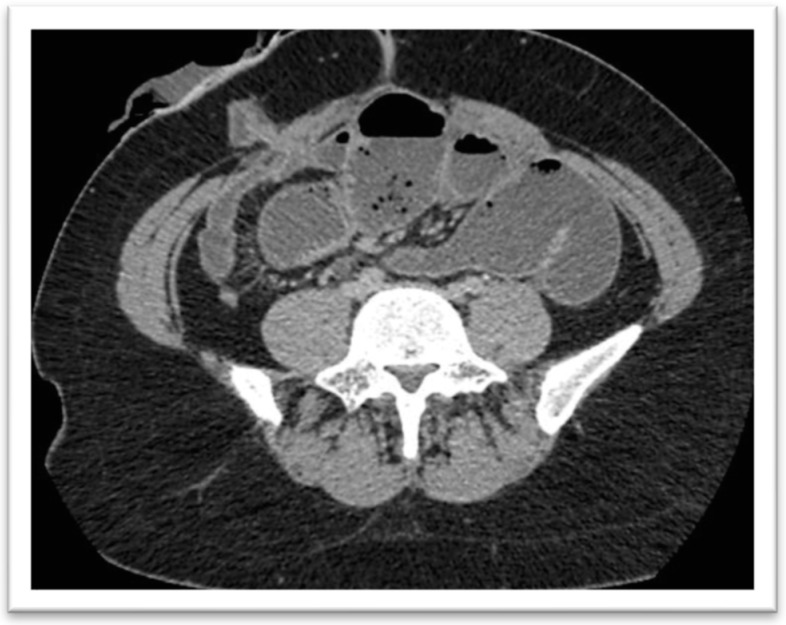
Abdominal CT scan demonstrating signs of small bowel obstruction in a patient with CD following proctocolectomy with end ileostomy.

The incidence of small bowel obstruction (SBO) in the context of proctocolectomy and end ileostomy for treating CD is unclear. However, it is believed to be high, as the procedure is complex, demands stoma formation, and requires extensive tissue manipulation, which can lead to the formation of adhesions. Adhesions that involve the small intestine and result in obstruction typically occur between the small bowel and the scar, between the small bowel and the site of surgery, and among the loops of the small bowel [53] (See Figure 2). Furthermore, the empty pelvic space that arises from proctectomy may become a location for trapping the small bowel, also generating SBO.

Minimally invasive surgery (MIS) is a helpful approach to reducing the incidence of SBO [52]. Ha and collaborators (2016) conducted a meta-analysis to assess the incidence of adhesion-related readmissions and surgery for adhesive SBO in patients who underwent colorectal surgery, and found that laparoscopic colorectal surgery significantly decreases the incidence of adhesive SBO and the rate of subsequent surgeries for adhesive SBO compared with open surgery [52]. MIS allows for precise dissection, minimal blood loss, and less bowel exposure to the environment during surgery, thereby theoretically reducing the failure of peritoneal tissue repair mechanisms and consequently reducing adhesion formation [52].

SBO can also indicate jejunoileal recurrence of the disease, which ultimately results in inflammatory or fibrotic stenosis. Symptoms include diminished or absent output from the ileostomy, recurrent episodes of abdominal pain, and vomiting [45]. Ecker and collaborators (2000) analyzed the reconstructions of the ileostomy in 92 patients who underwent colectomies. Approximately 30% of patients required ileostomy reconstruction during the follow-up period, and pre-stomal recurrence was the primary clinical indication in nearly 12% of cases [54].

## 6. Sexual and Urinary Dysfunction

Sexual function is likely to be impaired in patients with IBD, occurring more frequently in females than in males, with reported rates of 40–70% and 10–50%, respectively [55,56,57,58]. Sexual dysfunction in individuals with IBD can be attributed to several factors, including disease activity, surgical interventions, various medications, depression, and hypogonadism [56]. Active disease detrimentally impacts the overall quality of life, with debilitating symptoms like diarrhea, incontinence, perianal fistula discharge, and abdominal pain significantly hindering sexual function [56]. In fact, active perianal disease is a predictive factor of sexual dysfunction in female patients [59].

In the postoperative phase, sexual activity, particularly with a permanent ostomy, may be influenced by factors such as pain, feelings of unattractiveness due to scars and healing wounds, embarrassment regarding a stoma, or discomfort during intercourse [56]. Pelvic dissection can damage sympathetic and parasympathetic nerves or lead to scar tissue, which can impact sexual response. Specifically, injury to the autonomic nerves can lead to reduced vaginal lubrication, retrograde ejaculation, and erectile dysfunction [56].

Another issue associated with proctectomy in women is its detrimental impact on fertility. This is believed to result from significant surgical manipulation within the pelvis, which can cause adhesions and block the fallopian tubes. Key supporting structures, such as the pelvic floor, the lateral ligaments of the rectum, and the sphincter muscles, are closely connected to the female urogenital system. During proctectomy, these structures are partially or entirely excised, leading to unavoidable distortion and damage to the reproductive organs due to resulting postoperative adhesions and fibrosis. Radiological studies of pelvic changes in women following proctocolectomy demonstrate that most patients develop dorsal displacement of the vagina, as well as deformation and displacement of the uterus and adnexa towards the bottom of the pelvis [60]. Data from the Swedish National Patient Register demonstrated that women with IBD who underwent colectomy had a substantially lower fertility compared with the matched cohort from the general population [HR 0.65, CI 0.61–0.69]. Additionally, fertility seems to be dramatically reduced after completion proctectomy for any IBD subtype [HR 0.52, CI 0.47–0.58] [61].

Bladder dysfunction is another complication that may arise following pelvic colorectal surgery. Typically, this results from autonomic disruption during pelvic dissection, which leads to detrusor denervation and impaired bladder contractility. Most cases resolve within six months without long-term complications; however, patients should be monitored for developing small-capacity, poorly compliant, high-pressure bladders. Urinary dysfunction can result in additional harmful sequelae, including urinary tract infections, hydronephrosis, urinary reflux, pyelonephritis, and impaired renal function. Treatment and follow-up are tailored to each individual, taking into account urodynamic results, patient expectations, capabilities, and family dynamics support [62,63,64]. Neal and collaborators (1982) conducted a bladder function evaluation of 37 patients who underwent proctectomy for inflammatory bowel disease. They compared these patients with 34 control patients who underwent bowel resection without proctectomy. Symptoms of urinary dysfunction, particularly straining during micturition and a sensation of incomplete bladder emptying, were found to be significantly more common in patients after proctectomy than in the controls. Evidence of denervation of the bladder was observed in six patients after proctectomy, as indicated by the presence of capacious bladders with poor detrusor function. In contrast, none of the controls exhibited this finding. Additionally, the residual volume of urine in the bladder after micturition was significantly greater in patients after proctectomy compared to the controls [64].

## 7. CD Recurrence

Although proctectomy with a definitive stoma is deemed the definitive treatment for refractory CD colitis with anorectal involvement, patients and clinicians must still grapple with the specter of clinical recurrence following surgery [65]. The 20-year cumulative incidence of recurrence is estimated to reach 50% following proctocolectomy with end ileostomy, while the 15-year cumulative reoperation rate is approximately 25% [66,67,68]. Recurrence may occur in the proximal colon, small bowel, or even at the stoma, resulting in peristomal fistula, abscess, stenosis, obstruction, or bleeding. This phenomenon adversely impacts the quality of life of patients with CD and leads to a risk of further surgeries [65].

The risk factors for further disease progression differ across studies and are typically based on retrospective analyses of small cohorts from single centres. Some studies have demonstrated that patients with anorectal CD requiring proctectomy should undergo proctocolectomy with end-ileostomy rather than colostomy, regardless of whether proximal colonic involvement is absent. This is due to the fact that end-colostomy is linked with an early severe recurrence in the proximal colon and debilitating peristomal cutaneous lesions [47,65]. Other factors linked to clinical recurrence include male gender, the presence of anorectal lesions, prior small bowel surgery for CD, younger age at diagnosis, and small-bowel disease at the time of surgery [65,67,68,69].

Calvo and collaborators (2018) reported recurrence data for a cohort of 44 patients with CD who underwent total proctocolectomy with end ileostomy. They found an estimated clinical recurrence rate of 39% at 20 years, with the only risk factor for clinical recurrence being the presence of small bowel disease at the time of surgery [68]. Amiot and collaborators (2011) also analyzed data from a cohort of 55 patients, and clinical recurrence was significantly higher for patients with penetrating disease behavior [70]. Notably, they also described the absence of perianal disease as a risk factor for clinical recurrence, which seems paradoxical since perianal disease has traditionally been viewed as a marker of poor prognosis. Yamamoto and collaborators (1998) reported follow-up data from 103 patients with CD who underwent single-stage proctocolectomy. The 15-year cumulative reoperation rate for small bowel recurrence was 25 percent. Multivariate analysis revealed that male gender (HR, 2.4; *p* = 0.03) and age at operation of less than 30 years (HR, 2.6; *p* = 0.04) were the only risk factors for surgical recurrence [67].

During follow-up, clinical and endoscopic surveillance of patients undergoing proctocolectomy should be advisable to identify asymptomatic disease recurrence, guide early therapy, and avoid new surgeries [68]. At present, there are nearly no data to support prophylactic therapy for this group. However, through more extensive multicenter studies, criteria for effective risk stratification could be identified and validated, facilitating the creation of management algorithms specifically tailored for this population [71].

## 8. Conclusions

Long-term complications such as impaired perineal wound healing, stoma-related issues, sexual and urinary dysfunction, small bowel obstructions, and CD recurrence are common following proctectomy or proctocolectomy with definitive stoma. Those complications negatively affect quality of life and frequently necessitate further treatment. Patients should receive preoperative counselling regarding the implications of these particular issues, and regular follow-up must be guaranteed to identify any problems early, allowing for prompt treatment. Effective communication between the IBD multidisciplinary team and a personalized, patient-centered approach is key for successful surgical outcomes.

## 9. Future Directions

Complications following proctectomy for CD are frequent and not limited to non-healing perineal wounds. Future research must encompass the wide range of manifestations that can occur after proctectomy, generating more extensive series and multicentric data to ascertain which clinical factors are associated with a higher likelihood of complications. This may facilitate improved preoperative counselling and the prevention and early diagnosis of complications. It is crucial to emphasize the need to establish specialized centres in IBD surgery. Dedicated and multidisciplinary teams are likely to provide better outcomes.

The continual development of medical therapies is expected to lead to better disease control and prophylaxis of recurrence. Furthermore, the expansion and improvement of minimally invasive surgery are anticipated to enhance surgical outcomes. Minimal tissue manipulation is expected to lead to lower rates of adhesional obstruction. Additionally, improved visualization of the pelvis and precise dissection of anatomical structures may minimize tissue damage and inflammation, preserving sexual function, urinary health, and fertility.

## Data Availability

No new data were created or analyzed in this study. Data sharing does not apply to this article.
